# Biomarkers for Gynaecological Cancers

**DOI:** 10.3390/cancers18162689

**Published:** 2026-08-19

**Authors:** Apriliana E. R. Kartikasari

**Affiliations:** School of Health and Biomedical Sciences, Royal Melbourne Institute of Technology (RMIT) University, Bundoora, VIC 3082, Australia; april.kartikasari@rmit.edu.au

Gynaecological cancers, encompassing diseases with profoundly different biological behaviours, clinical trajectories, and therapeutic needs, remain a major global health challenge. Endometrial, ovarian, cervical, and vulvar cancers collectively account for a substantial burden of morbidity and mortality among women worldwide. Yet, despite considerable advances in surgery, systemic therapies, molecular classification, and screening, one central challenge persists: identifying clinically significant disease early and accurately enough to alter its course.

The studies presented in this collection provide important insights into the evolving field of gynaecological oncology. The molecular, elemental, metabolic, epigenetic, and protein landscapes of tumours are subject to increasing scrutiny. Together, these studies point towards a future in which diagnosis and prognosis are increasingly individualised, biologically informed, and minimally invasive.

Changes in elemental homeostasis in endometrial and ovarian cancers constitute a particularly intriguing area. Ordon et al. [1] demonstrated that higher-grade endometrial tumours were associated with increases in calcium, phosphorus, magnesium, manganese, sodium, and potassium, whereas sodium and potassium showed a tendency to decrease. A similar trend was observed in ovarian tumours [1]. Importantly, histological grade emerged as the principal determinant of these elemental alterations, whereas factors such as diabetes, smoking, menopausal status, age, and body mass index had more limited or inconsistent effects. Elements such as magnesium, manganese, calcium, and phosphorus are known to participate in numerous cellular processes, including energy metabolism, signalling, proliferation, and oxidative stress regulation [2]. Alterations in their intracellular or tissue distribution could therefore reflect the metabolic reprogramming characteristic of cancer. Elemental profiling may ultimately become a complementary tool for understanding tumour biology, although its clinical utility requires validation in larger, independent populations. An important question for future research is whether elemental signatures can distinguish tumour subtypes, predict recurrence, or provide information beyond molecular classification. Combining elemental profiles with genomic, metabolomic, and pathological data may ultimately prove more informative than measuring elemental profiles in isolation.

Molecular characterisation of cancers provides another powerful route toward precision oncology. An investigation of mixed and mixed-feature endometrial carcinomas illustrates why conventional histological classification alone may be insufficient [3]. These tumours, frequently containing serous and endometrioid components, were associated with older age, high-grade morphology, and poorer disease-free survival than pure endometrioid carcinomas. More importantly, molecular analysis revealed evidence of a shared clonal origin, including identical TP53 and PIK3CA mutations across different histological components. The presence of ERBB2 amplification, which was more frequently observed in mixed endometrial tumours than in pure carcinomas [3,4], suggests that this genetic alteration may distinguish mixed tumours from their pure carcinoma counterparts. The role of these signature mutations in the pathogenesis of mixed endometrial carcinoma and their potential as therapeutic targets warrant further investigation.

Histopathology itself is also evolving. An investigation of tumour budding and poorly defined clusters in vulvar squamous cell carcinoma [5] demonstrates how subtle morphological features can provide prognostic information. The association between tumour budding and metastasis, recurrence, and overall survival suggests that seemingly small architectural details may capture aggressive tumour behaviour. These findings support continued efforts to identify reproducible pathological biomarkers that can complement conventional staging and grading.

The need for improved diagnostic biomarkers is equally evident in regard to cervical disease. Although most cervical intraepithelial neoplasia lesions regress, a subset persists or progresses to invasive cancer, and conventional assessment cannot reliably predict which lesions will progress. The study examining ZNF671 methylation [6] as part of a broader panel of cervical-cancer-associated methylation markers [7] offers a promising approach. ZNF671 methylation increased with lesion grade and was particularly associated with progressive disease. Such biomarkers, capable of distinguishing lesions likely to progress from those likely to regress, could reduce unnecessary treatment while enabling closer surveillance of women at higher risk.

Identifying minimally invasive biomarkers and developing accessible technologies for their detection are essential for improving equitable cancer detection and management, particularly in rural, remote, and low-income communities, where limited access to vaccination, screening, diagnostic infrastructure, and treatment contributes substantially to cancer mortality [8]. In this issue, Habbani et al. [9] developed a simple method for detecting proteins, including TOP2A, MCM2, VCP, and p16INK4a, in cervical swabs, demonstrating their potential for cervical cancer screening. The ability to quantify these proteins from cervical specimens and distinguish clinically relevant disease categories creates opportunities for developing simpler, more accessible point-of-care approaches.

Similarly, the detection of a three-marker protein panel comprising PAX2, PTEN, and β-catenin in secretory endometrium addresses a challenging diagnostic problem in detecting endometrial precancer [10]. Secretory changes can obscure the morphological features of histological precursors to endometrial carcinoma. The cited study demonstrated that this three-marker panel can aid identification of endometrial precancerous lesions in clinical practice, particularly in diagnostically challenging secretory-phase endometria.

Finally, ovarian cancer illustrates perhaps the greatest unmet need for early detection. Most patients are diagnosed at advanced stages, when curative treatment becomes substantially more difficult. Circulating microRNAs offer an appealing approach because they can be measured through minimally invasive blood tests. Meta-analytic evidence [11] indicates that panels of circulating miRNAs perform better than individual miRNAs, and combinations with established biomarkers such as CA-125 and HE4 can further improve diagnostic accuracy. CA-125 and HE4 remain clinically useful, but neither alone provides the sensitivity and specificity required for reliable early detection [12]. This issue has driven growing interest in liquid biopsy approaches, including circulating tumour DNA, microRNAs, extracellular vesicles, DNA methylation, and multi-analyte signatures. Importantly, distinct miRNA patterns appear to have the potential for stage-specific detection, raising the possibility of not only identifying ovarian cancer but also providing information about disease progression.

Additionally, a systematic review of 128 gynaecological cancer studies identified genetic variants within DNA repair pathways, particularly Base Excision Repair (BER) and Nucleotide Excision Repair (NER), as promising biomarkers for assessing gynaecological cancer risk [13]. These variants may influence DNA repair efficiency and individual susceptibility, providing opportunities for earlier risk assessment and supporting the early detection of gynaecological cancers.

In summary, the future of gynaecological oncology is therefore unlikely to depend on a single revolutionary biomarker; it will likely depend on the convergence of multiple forms of biological information to enable more accurate diagnosis, prognosis, treatment selection, and monitoring. Equally important will be the development of minimally invasive, affordable, and accessible detection technologies that can be implemented across diverse healthcare settings, including in rural, remote, and low-income communities. The ultimate goal is to identify cancer earlier, distinguish women who require immediate treatment from those who can be safely monitored, predict tumour aggressiveness and recurrence, and match therapy to the biology of each individual cancer. Together, these advances could reduce disparities in cancer care, improve survival, and move this field towards the long-term goal of preventing and ultimately eliminating deaths caused by gynaecological cancers.

## References

[1] Ordon P., Boroń K., Bereza K., Boroń D., Ossowski P., Sirek T., Sirek A., Kulej W., Wyrobiec G., Grabarek B.O. (2026). Assessment of essential and toxic element levels in endometrial and ovarian cancer. Cancers.

[2] Razzaque M.S., Wimalawansa S.J. (2025). Minerals and human health: From deficiency to toxicity. Nutrients.

[3] Bhardwaj S., Saleh M., Kinoshita Y., Brody R., Lukatskaya O., Blank S.V., Baskovich B., Kalir T. (2026). Endometrial Mixed and Mixed-Feature Carcinomas: Small Cohort Clinicopathologic and Molecular Studies. Cancers.

[4] Haight P.J., Esnakula A., Riedinger C.J., Suarez A.A., Gillespie J., Patton A., Chassen A., Cohn D.E., Cosgrove C.M. (2025). Molecular characterization of mixed-histology endometrial carcinoma provides prognostic and therapeutic value over morphologic findings. npj Precis. Oncol..

[5] Klamminger G.G., Bitterlich A., Haj Hamoud B., Solomayer E.-F., Ertz M., Schnöder L., Holleczek B., Brenner W., Hasenburg A., Wagner M. (2025). Evaluation of tumor budding and poorly defined clusters as histological biomarkers in squamous cell carcinomas of the vulva. Cancers.

[6] Dübbel L., Göken-Riebisch A., Knoll K., Hippe J., Marticke C., Schild-Suhren M., Malik E. (2025). The DNA methylation marker ZNF671 has prognostic value for progressing cervical intraepithelial neoplasia. Cancers.

[7] Ren Y., Qin F., Shen L., Li L., Wu Q., Yi P. (2025). Triage of women with a positive HPV DNA test: Evaluating a DNA methylation panel for detecting cervical intraepithelial neoplasia grade 3 and cervical cancer in cervical cytology samples. BMC Cancer.

[8] World Health Organization (2024). WHO Guideline for Screening and Treatment of Cervical Pre-Cancer Lesions for Cervical Cancer Prevention: Use of Dual-Stain Cytology to Triage Women After a Positive Test for Human Papillomavirus (HPV).

[9] Habbani S.F., Dowlatshahi S., Lichti N., Broman M., Tecle L., Bolton S., Flowers L., Guerrero-Preston R., Linnes J.C., Mohammed S.I. (2025). Protein biomarkers enable sensitive and specific cervical intraepithelial neoplasia (CIN) II/III+ detection: One step closer to universal cervical cancer screening. Cancers.

[10] Niu S., Molberg K., Chen J., Conrad L., Lucas E., Chen H. (2025). Expression characteristics of 3-marker panel (PAX2, PTEN, and β-catenin) in benign interval and secretory endometrium and secretory endometrial precancer. Cancers.

[11] Kartikasari A.E.R., Michel-Lara P., Exton H., Tekin-Sari K., Alnefai E.M.M., Mitchell A., Sanchez-Huertas C., Plebanski M. (2024). Circulating microRNAs as Diagnostic Biomarkers to Detect Specific Stages of Ovarian Cancer: A Comprehensive Meta-Analysis. Cancers.

[12] Zhang R., Siu M.K., Ngan H.Y., Chan K.K. (2022). Molecular biomarkers for the early detection of ovarian cancer. Int. J. Mol. Sci..

[13] Szatkowska M., Zdrada-Nowak J. (2025). Genetic polymorphisms in base excision repair (BER) and nucleotide excision repair (NER) pathways as potential biomarkers for gynecological cancers: A comprehensive literature review. Cancers.

